# Composite Coating of Oleaster Gum Containing Cuminal Keeps Postharvest Quality of Cherry Tomatoes by Reducing Respiration and Potentiating Antioxidant System

**DOI:** 10.3390/foods13101542

**Published:** 2024-05-15

**Authors:** Ruojun Ding, Xishuang Dai, Zhong Zhang, Yang Bi, Dov Prusky

**Affiliations:** 1 College of Food Science and Engineering, Gansu Agricultural University, Lanzhou 730070, China; dingrj@gsau.edu.cn (R.D.); daixs2024@163.com (X.D.); biyang@gsau.edu.cn (Y.B.); dovprusk@agri.gov.il (D.P.); 2 Department of Postharvest Science of Fresh Produce, Agricultural Research Organization, The 12 Volcani Center, Beit Dagan 50200, Israel

**Keywords:** oleaster gum, cuminal, cherry tomato, fruit quality, antioxidant system

## Abstract

Exploring the green and affordable protection of perishable cherry tomato fruits during storage, herein, the protective efficacy, and its underpinning mechanisms, of a coating of oleaster gum, alone or incorporated with cuminal, on cherry tomatoes stored at ambient temperature was investigated. The composite coating of oleaster gum with 0.1% cuminal reduced the decay, respiration rate, weight loss, and softening of the fruits and decelerated the decreases in their total soluble solid, titratable acidity, and soluble protein levels, and therefore maintained their marketability. Furthermore, it reduced the accumulation of $O_{2}^{\cdot -}$ and H_2_O_2_ in the fruits and mitigated cell membrane lipid oxidation and permeabilization, thereby retarding their senescence. Instrumentally, it elevated the activities of superoxide dismutase, catalase, peroxidase, and ascorbate peroxidase and the levels of ascorbic acid and glutathione. This potentiation of the fruits’ antioxidant system makes this composite coating a promising approach to keeping the postharvest quality of perishable fruits.

## 1. Introduction

Most fruits and vegetables suffer severe postharvest quality loss and are susceptible to microbial infestation during storage [1]. Cherry tomato *(Solanum lycopersicum* var. *cerasiforme*) fruits are globally popular owing to their attractive appearance, unique flavor, and juicy texture [2], as well as being rich in vitamins, minerals, and bioactive compounds like carotenoids and flavonoids [3]. The fruits can be consumed fresh or processed, such as in sauces, puree, ketchup, and more [4]. Nevertheless, being climacteric, the fruits are highly perishable and have a short postharvest life [5]. Fast physio-biochemical processes like transpiration, respiration, and ethylene emission accelerate the fruits’ ripening, rendering them susceptible to phytopathogenic attacks and significantly reducing their marketability and nutritional quality [6]. Meanwhile, consumers always expect fruits to have a relatively long shelf-life with maximal freshness.

Cold storage can mitigate the loss of freshness and extend the shelf-life of cherry tomatoes by reducing their respiration and minimizing microbial growth, but it often causes chilling injury to the fruit [7]. Although several other techniques like hypobaric storage in a high relative humidity, modified-atmosphere packaging, atmosphere controlling, and sanitizing treatments (using UV-C, ozone, chemical sanitizers, and natural antimicrobials, among others) have been used to extend the shelf life of these fruits, they are expensive to apply on an industrial scale or can leave detrimental residues often rejected by informed consumers [8,9]. Fruit coating is an affordable technology to prolong the postharvest life of fruit and reduce their quality deterioration [10].

Among diverse coating formulae, natural gum-based ones have garnered considerable attention in fruit preservation by virtue of their sustainability, convenience, and versatility when compared with synthetic packaging [11]. These competitive advantages have been demonstrated in a wide array of fruit and vegetables including tomato [2], strawberry [12], apricot [13], mushroom [14], and guava [15]. Exuded from the stems of olive trees (*Elaeagnus angustifolia* L.) of the Elaeagnacea (*Araliaceae*) family, oleaster gum (OG) consists of heteropolysaccharides with motifs of various monosaccharides such as arabinose, galactose, rhamnose, and mannose and their derivatives like uronic acid [16,17]. Auspiciously, OG demonstrates antioxidant, antimicrobial, anti-inflammatory, and anti-cancer efficacy in various backgrounds. With a hydration capacity like that of gum arabic, OG can form a gel and stabilize emulsion in water [18]. Together with other types of botanical extracts, these attributes can be employed to formulate bioactive coatings to achieve a promising potency in postharvest protection [19].

Essential oils possess a wide spectrum of bioactivity, notably, including antimicrobial and antioxidant activities, and can be added to hydrocolloids as natural food preservatives [1]. Cuminal (CA), a dominating component of cumin essential oil, is widely used in food, medicine, and fragrance cosmetics [20,21]. CA has noteworthy antimicrobial [22], anti-toxigenic [23], and anti-neoplastic [24] properties in various application situations. When capsuled in porous starch, it forms stable hydrogen bonds with the oxygens of the carbohydrate hydroxyl-2,3 [25], and this interaction overcomes certain drawbacks such as hydrophobicity and fast volatility that impede its sustainable release and function. Sharing some structural similarities with, but being more hydrophilic and tensile than, starch, OG is also a potential matrix to establish a slow-release system for CA, but this potential has not been investigated. Moreover, in postharvest handling, to our knowledge, the employment of OG alone or together with CA in the preservation of fresh fruit and vegetables has not been reported.

Herein, the efficacy of an OG-based coating with or without the incorporation of CA in keeping the postharvest quality of cherry tomatoes during room temperature storage was assessed together with the investigation of the underpinning mechanisms.

## 2. Materials and Methods

### 2.1. Materials

Cherry tomatoes were harvested without pesticides in a high-efficiency greenhouse at the Yongjing Industrial Cooperation Demonstration Park in Gansu, China, and brought immediately to the Postharvest Laboratory of Gansu Agricultural University. Fruits with a uniform shape, size, color, and maturity, and free from mechanical injuries and microbial infestation, were selected for this experiment.

OG was procured from trees of *E. angustifolia* L. in the Gobi Desert of Hetian, Xinjiang, China, and dried in air. CA (CAS # 122-03-2) with a purity of ≥97% was purchased from Macklin Biotechnology, Shanghai, China.

### 2.2. Methods

#### 2.2.1. Preparation of Coating Solutions

According to the method published in [13,18] with some modifications, we crushed the OG and sifted it through a 100-mesh sieve after removing visible impurities. The obtained OG powder (2 g) was dissolved in sterilized distilled water (100 mL) and agitated for 2 h at 45 °C with a magnetic stirrer. As a plasticizer to improve the strength and pliability of the coating, glycerol was added (1%, *v*/*v*) to the OG solution, which was cooled to 20 °C. A CA emulsion in Tween 80 (0.1%, *v*/*v*) was mixed with the above solution to obtain the final coating solutions with 1% (mg/mL) OG and varying concentrations of CA (0, 0.1, and 0.2%, *v*/*v*). All the coating solutions were sonicated for 20 min at 20 °C and 50% amplitude to defoam and degas them. The coating solutions were kept static until application.

#### 2.2.2. Coating Treatment

The coating approaches were tailored according to the specific characteristics of the fruits. The fruits were cleaned with tap water and dried in air. The fruits were dipped in the coating solutions (using distilled water as the control) for about 30 s [26] and dried in air. The fruits were divided into four groups (control, OG, OG + 0.1% CA, and OG + 0.2% CA). Each group had three replicates, wherein each replicate had 50 fruits. All fruits were packed accordingly in plastic crispers and stored at 25 °C and 85~95% RH [27]. Samples were randomly taken from each group at 0, 3, 6, 9, 12, and 15 days after storage for analyses.

#### 2.2.3. Evaluation of Decay Index and Marketability

The decay index and marketability of the fruits were assessed according to the previous method with slight modifications [7]. The decay index was defined as the percentage of fruits exhibiting decay symptoms such as mold spots or rot in each group. The fungal growth symptoms on the fruit surfaces were visually evaluated using a scale, where 0 = no symptoms of decay, 1 = 1~10%, 2 = 11~25%, 3 = 26~50%, 4 = 50~75%, and 5 ≥ 75% decay. The decay index was calculated using the following formula:

$$\mathrm{Decay}\;\mathrm{index}=\frac{\sum \;(\mathrm{decay}\;\mathrm{scale})\;\times \;(\mathrm{Number}\;\mathrm{of}\;\mathrm{fruit}\;\mathrm{at}\;\mathrm{each}\;\mathrm{scale})}{\mathrm{Total}\;\mathrm{number}\;\mathrm{of}\;\mathrm{fruit}\;\times 5\;}\times \;100\%$$

The levels of color, aroma, and shriveling of the fruits were organoleptically determined as a whole with a 1–5 rating scale, where 1 = unusable, 2 = usable, 3 = fair, 4 = good, and 5 = excellent. Fruits receiving ratings of 4 or above were deemed marketable and counted to calculate the marketability of the fruits via the formula below:

$$\mathrm{Percentage}\;\mathrm{marketability}=\frac{\mathrm{Number}\;\mathrm{of}\;\mathrm{marketable}\;\mathrm{fruit}\;}{\mathrm{Total}\;\mathrm{of}\;\mathrm{fruit}}\times \;100\%$$

#### 2.2.4. Measurement of Respiration Rate, Weight Loss, and Firmness

The fruit respiration rate was determined by detecting the CO_2_ production via an infrared CO_2_ determinator (JFQ-3150H, Jun-Fang-Li-Hua Technology-Research Institute, Beijing, China), and the results were expressed in mg kg^−1^ h^−1^.

The fruits in each group were weighed at 0, 3, 6, 9, 12, and 15 d during storage. Weight loss was calculated as the difference between weights at initial and specific time points divided by the initial weight.

Fruit firmness was measured via a fruit firmness analyzer (GY-4, TOP Instrument, Hangzhou, China) with a 3 mm probe at three different positions on the fruit equator, and the results were expressed in Newtons (N).

#### 2.2.5. Determination of Total Soluble Solid (TSS), Titratable Acidity (TA), and Soluble Protein Levels

The TSS content (%) was analyzed as Brix with a digital refractometer (PR-32, Atago, Tokyo, Japan) calibrated with distilled water [27].

TA (%) was assessed by titrating samples with 0.1 N NaOH. The results were reported as g citric acid equivalents per 100 g of fresh weight [4], wherein the acid factor of citric acid is 0.064.

Determined by the Coomassie brilliant blue G-250 method, the total soluble protein content was expressed in mg kg^−1^ on a fresh weight basis [28].

#### 2.2.6. Determination of Membrane Permeability and Malondialdehyde (MDA) Content

Cell membrane permeability was determined with the previous operations after minor modifications [29]. The fruit slices (10 g) were placed in 40 mL of deionized water and incubated at 25 °C, and their conductivity was determined at 0 and 3 h with a conductometer (DDS-307A, Ridao, Shanghai, China) and recorded as E_0_ and E_1_, respectively. The samples were transferred to boiling water for incubation for 0.5 h and then cooled down immediately to 25 °C. The conductivity was recorded as E_2_. The cell membrane permeability was calculated with the following formula:

$$\mathrm{Cell}\;\mathrm{membrane}\;\mathrm{permeability}=\frac{E_{1}-E_{0}}{E_{2}}\times \;100\%$$

The MDA content was determined according to the relevant methods [29]. Fruit flesh (3 g) was homogenized in 6 mL of precooled trichloroacetic acid (TCA) and centrifuged for 20 min at 12,000× *g* at 4 °C. The supernatant (2 mL) was reacted with 2 mL of 0.67% (*w*/*v*) 2-thiobarbituric acid, and the absorbance was determined at 450, 532, and 600 nm after 20 min of incubation in boiling water. The MDA content was calculated with the following formulae and expressed in mol kg^−1^:

C_MDA_ (µmol L^−1^) = 6.45 × (OD_532_ − OD_600_) − 0.56 × OD_450_

$$\mathrm{MDA}\;\mathrm{content}\;(\mathrm{mol}\;{\mathrm{kg}}^{-1})=\frac{C_{MDA}\;\times \;\mathrm{Extraction}\;\mathrm{volume}\;}{\mathrm{Fruit}\;\mathrm{fresh}\;\mathrm{weight}}\times \;100\%$$

#### 2.2.7. Determination of $O_{2}^{\cdot -}$ and H_2_O_2_

The $O_{2}^{\cdot -}$ content was quantified using an established scientific protocol [30]. Briefly, 5 g of fruit flesh was homogenized in 15 mL of 60 mmol L^−1^ phosphate buffer (pH 7.8) at 4 °C for 15 min. The supernatant (1 mL) was mixed with 0.1 mL of 10 mmol L^−1^ hydroxylamine hydrochloride and kept at 37 °C for 20 min. The absorbance at 530 nm was measured. The standard curve of NaNO_2_ was used to calculate the $O_{2}^{\cdot -}$ content in μmol per gram of fresh weight.

The H_2_O_2_ content was evaluated based on a previous approach [31]. Concisely, fruit flesh (5 g) was homogenized in 50 mL of 5% TCA (*w*/*v*) at 4 °C and centrifuged at 10,000× *g* at 4 °C for 15 min. The supernatant (0.5 mL) was mixed with 0.5 mL of 10 mmol L^−1^ K_3_PO_4_ buffer (pH 7.0) and 1 mL of a 1 mol L^−1^ KI solution, and the 390 nm absorbance was tested using a UV spectrophotometer (UV-754, Precision & Scientific Instrument, Shanghai, China). The H_2_O_2_ content was calculated via the standard curve of analytical-grade pure H_2_O_2_ dissolved in acetone, and the results were expressed in mmol kg^−1^.

#### 2.2.8. Analyses of Antioxidant Enzyme Activities

At 4 °C, fruit flesh (5 g) was homogenized in 20 mL of 50 mmol L^−1^ phosphate buffer (pH 7.8) containing 1.0 mmol L^−1^ EDTA, 0.3% (*v*/*v*) Triton X-100(Targetmol, Shanghai, China), and 2% (*w*/*v*) polyvinylpyrrolidone (PVP). The solution was centrifuged at 10,000× *g* for 20 min, and the supernatant was collected for assays of the enzyme activities.

The superoxide dismutase (SOD) activities were assayed as described in a previous study [32]. One unit of SOD activity was defined as the amount of the enzyme needed to inhibit 50% nitroblue tetrazolium reduction tested at 560 nm and expressed in U kg^−1^.

The catalase (CAT) activities were assayed following the method previously described in [33] with slight modifications. The reaction mixture consisted of 0.1 mL of the enzyme extract and 2.9 mL of 20 mmol L^−1^ H_2_O_2_. The absorbance at 240 nm was recorded for 3 min at 30 s intervals, with distilled water as a blank reference. One unit of CAT activity was defined as a decrease of 0.01 per minute in the 240 nm absorbance and expressed in U kg^−1^.

The peroxidase (POD) activities were determined following a methodology with slight modifications [34]. The reaction mixture consisted of 1 mL of 50 mmol L^−1^ phosphate buffer (pH 5.5), 1 mL of 25 mmol L^−1^ guaiacol, 0.5 mL of 2% (*v*/*v*) H_2_O_2_, and 0.5 mL of the enzyme extract. The absorbance at 470 nm was recorded at 30 s intervals for 3 min, with distilled water as a blank reference. One unit of POD activity was defined as an increase of 0.01 per minute in the 470 nm absorbance, and the results were expressed in U kg^−1^.

The ascorbate peroxidase (APX) activities were measured according to the relevant method [35] with slight modifications. The reaction mixture included 0.1 mL of the enzyme extract, 2.6 mL of 50 mmol L^−1^ phosphate buffer (pH 7.0), and 0.3 mL of 30% (*v*/*v*) H_2_O_2_. The absorbance at 290 nm was recorded at 30 s intervals for 3 min, with distilled water as a blank reference. One unit of APX activity was defined as a decrease of 0.01 per minute in the 290 nm absorbance, and the results were expressed in U kg^−1^.

#### 2.2.9. Determination of Ascorbic Acid (AsA) and Glutathione (GSH) Levels

The AsA content was assayed by the 2,6-dichlorophenolindophenol method [36]. Briefly, fruit flesh (5 g) was homogenized in 10 mL of 20 g L^−1^ oxalic acid, and the solution volume was adjusted to 100 mL with the oxalic acid. After 20 min of centrifugation at 8000× *g*, 10 mL of the supernatant was collected and titrated with 2,6-dichlorophenolindophenol until the pink color lasted for more than 15 s, and the concentrations were expressed in mg kg^−1^.

The GSH content was measured following a previous method [37] with slight modifications. Fruit flesh (5 g) was homogenized in 10 mL of precooled 5% (*w*/*v*) TCA containing 5 mmol L^−1^ EDTA-Na_2_ and centrifuged at 8000× *g* for 20 min at 4 °C. The reaction mixture containing 1 mL of the enzyme extract, 1 mL of sodium phosphate buffer (100 mmol L^−1^, pH 7.6), and 0.5 mL of 4 mmol L^−1^ dithionitrobenzoic acid was incubated at 25 °C for 10 min, and the 412 nm absorbance was measured. The GSH content was calculated via the standard curve of GSH, and the results were expressed in mmol kg^−1^.

### 2.3. Statistical Analysis

The results were expressed as the means ± standard error (SE) of the experiments performed in triplicate. The data were analyzed by SPSS 25 and plotted with Origin 8.5, and Duncan’s multiple test at *p* < 0.05 was conducted.

## 3. Results

### 3.1. Fruit Decay Index and Marketability

**Figure 1:**
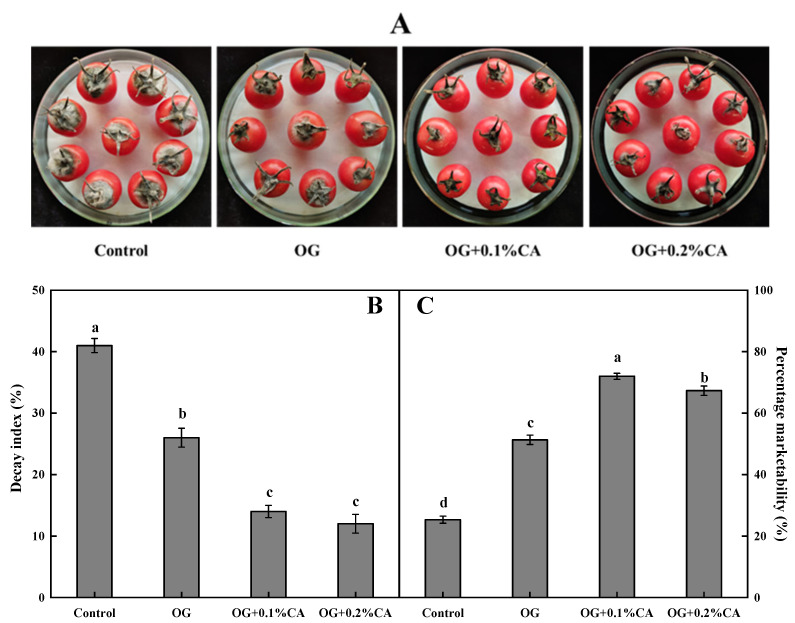
Visual appearance (**A**), decay index (**B**), and percentage marketability (**C**) of cherry tomatoes without coating and coated with OG, OG + 0.1% CA and OG + 0.2% CA after 15 d of storage at 25 °C and 85~95% RH. Bars represent standard errors (±SE). Different lowercase letters mean statistically significant differences (*p* < 0.05).

Microbial spoilage and physiological decay affect the appearance and marketability of cherry tomatoes. After 15 d of storage at 25 ± 1 °C, the levels of visible mold growth in coated fruits were significantly lower than those in fruits without a coating (Figure 1A). The coatings significantly reduced the decay index (Figure 1B) and improved the marketability (Figure 1C) of the fruits, and both the composite coatings with CA were more effective than the plain coating of OG alone. Among them, the fruits with the composite coating with OG and 0.1% CA showed the best appearance and marketability. Notably, among the composite coatings, doubling the CA concentration showed no further decrease in the decay index but, unexpectedly, reduced the fruit marketability. Thus, the OG + 0.1% CA formula was selected for further analyses. OG + 0.2% CA will not be discussed anymore due to its undistinguished performance compared with OG + 0.1% CA and the special smell of cuminal.

### 3.2. Respiration Rate, Weight Loss, and Firmness

**Figure 2:**
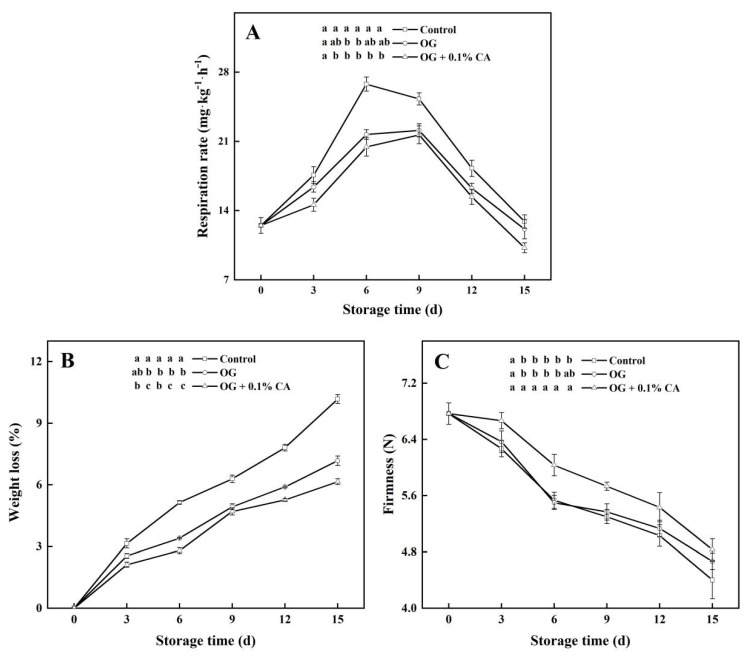
Composite coating of OG and 0.1% CA delayed and significantly lowered respiration peak (**A**) and mitigated the fruit weight loss (**B**) and loss of firmness (**C**) of the cherry tomatoes stored at 25 °C and 85~95% RH. Bars represent standard errors (±SE). Different lowercase letters mean statistically significant differences (*p* < 0.05).

The respiration rate is closely connected to the senescence of postharvest fruits. The respiration rate of all the fruits first increased and then decreased during storage (Figure 2A). Respiration peaks were observed on day 6 in the fruits without a coating and on day 9 in the coated fruits, suggesting a peak delay attendant on the coating. At around days 6 to 9, the fruits without a coating demonstrated significantly higher respiration rates than those coated. The fruits with the composite coating showed the lowest respiration rate among all fruits from day 0 onward. On day 15, the respiratory rate of the fruits with the composite coating was 20.56% and 15.58% lower than those of the fruits with no coating and the plain coating, respectively, and 18.08% lower than those of all fruits on initial day 0. In short, the coatings reduced the respiration and also delayed the respiration peak of the fruits.

Changes in and the severity of weight loss reflect the quality deterioration intensity of fruits. Herein, the weight loss of all the fruits continuously increased during storage, and from day 6 onward, the data of the coated fruits were significantly lower than those of the fruits without a coating. The fruits with the composite coating showed the lowest weight loss among the samples at most storage points (Figure 2B). The weight loss of postharvest tomatoes is mainly related to their respiration and transpiration rates. Notably, the weight loss of the coated fruits at day 15 was significantly lower than that of the fruits without a coating, wherein the fruits with the composite coating showed the lowest weight loss (6.15%).

Firmness reflects the ripening and softening states of postharvest fruits. Herein, the firmness of all the fruits decreased during storage; however, the fruits with the composite coating showed a significantly higher firmness at most of the storage time points (Figure 2C). In contrast, no significant difference in firmness was observed between the fruits with the plain coating and without a coating. Notably, the firmness of all the fruits steadily reduced before day 12 but slumped thereafter. On day 15, the fruits with the composite coating had the highest firmness of 4.8 N among all the fruits.

### 3.3. TSS, TA, and Soluble Protein Levels

**Figure 3:**
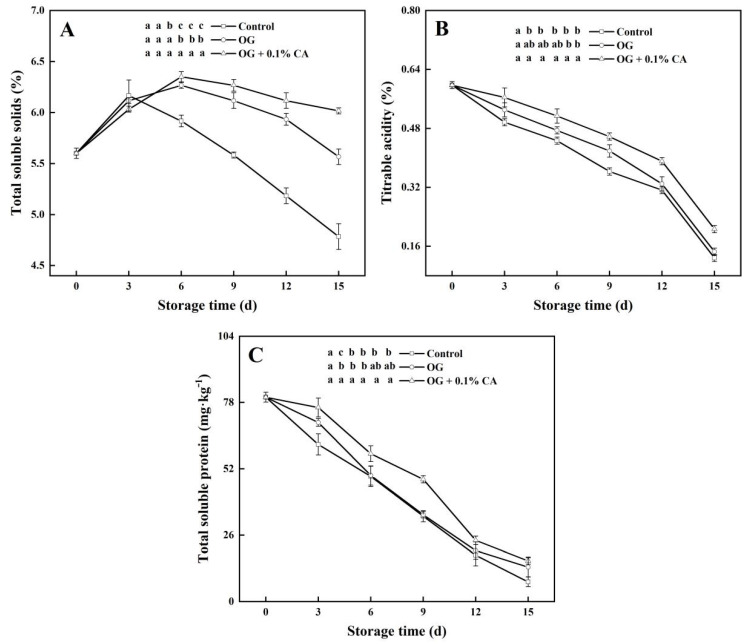
Composite coating of OG and 0.1% CA retarded the declines in TSS (**A**), TA (**B**), and total soluble protein (**C**) levels in cherry tomatoes stored at 25 °C and 85~95% RH. Bars represent standard errors (±SE). Different lowercase letters mean statistically significant differences (*p* < 0.05).

The TSS content is an important attribute of the edible qualities of fruits. The TSS contents of all the fruits increased in the first 3 d of storage and then diverged with a steady decline in the fruits without a coating and further rose until day 6 in the coated fruits (Figure 3A). From day 9 onward, significant differences existed in the TSS contents between the fruit groups, wherein the fruits with the composite coating had the highest (6.02%) TSS content followed by those with the plain coating (5.57%), and those without a coating (4.78%).

TA is important in determining fruit taste, flavor, and sensory qualities. Here, the TA of all the fruits steadily decreased before day 12 and subsequently experienced a slump (Figure 3B); nonetheless, the fruits with the composite coating always showed a significantly higher TA than the others. On day 15, the highest TA was found in the fruits with the composite coating (0.21%), followed by the TA values of 0.15 and 0.12% found in the fruits with the plain coating and without a coating, respectively.

Soluble proteins in plant cells are mainly enzymes involved in various metabolisms and stress resistance [28]. The total soluble protein contents in all the fruits decreased during storage. Significant differences existed between the control and composite coating groups. However, no significant difference was observed between the control and plain coating groups from day 6 onward (Figure 3C). On day 15, the highest total soluble protein content was observed in the composite coating group (15.89 mg kg^−1^), followed by the plain coating group (13.56 mg kg^−1^), and the control group (0.12 mg kg^−1^).

### 3.4. Membrane Permeability and MDA Content

**Figure 4:**
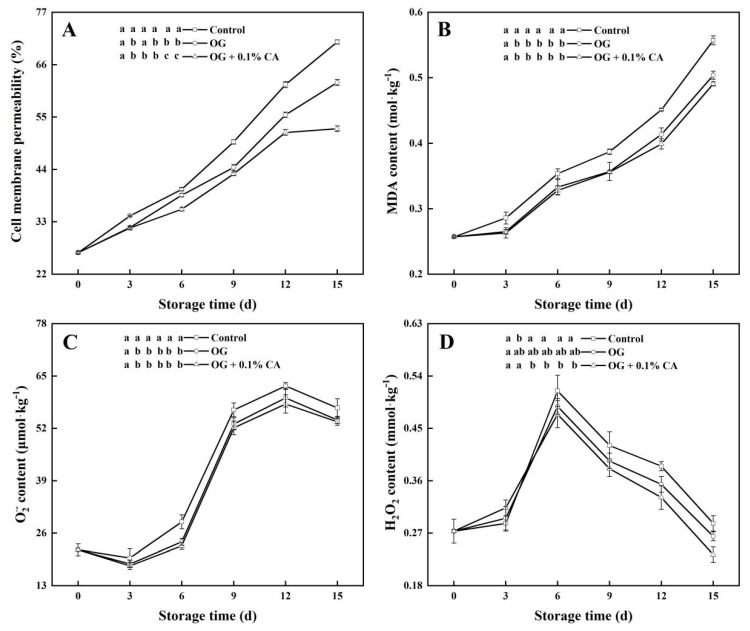
Composite coating of OG and 0.1% CA mitigated cell membrane permeabilization (**A**), reduced MDA production (**B**), and lowered $O_{2}^{\cdot -}$ (**C**) and H_2_O_2_ (**D**) generation in cherry tomatoes stored at 25 °C and 85~95% RH. Bars represent standard errors (±SE). Different lowercase letters mean statistically significant differences (*p* < 0.05).

Membrane permeability reflects the integrity or damage severity of the cellular membrane [32] and, in plant cells, is generally determined by analyzing the relative electrical conductivity of plant tissues. Here, the membrane permeability of all fruits increased during storage (Figure 4A), and it increased faster in the fruits without a coating than in the coated fruits, suggesting that the coatings retarded the elevation in the cell membrane permeability of the fruits. On day 15, the fruits with the composite coating showed the lowest membrane permeability (52.56%) among all fruits, and significant differences were observed between the composite coating group and the other groups, illustrating that the composite coating reduced damage to the membrane integrity and delayed the senescence of the cherry tomatoes during storage. The over-accumulation of MDA oxidatively damaged the cellular membrane system and resulted in an increase in membrane permeability (Figure 4B). The MDA contents in all the fruits gradually increased, but those in the coated groups were significantly lower than their counterpart data for the control. On day 15, the composite coating group had the lowest MDA content (49.06 mol kg^−1^), and significant differences were observed between the coated and control groups.

### 3.5. $O_{2}^{\cdot -}$ and H_2_O_2_ Contents

$O_{2}^{\cdot -}$ and H_2_O_2_ are important intracellular reactive oxygen species (ROS), and their excessive accumulation irreversibly damages cells [38]. Here, the $O_{2}^{\cdot -}$ content demonstrated an S-shaped curve during storage in all groups, but the highest concentrations of $O_{2}^{\cdot -}$ were consistently observed in the control group (Figure 4C). On day 15, the level of $O_{2}^{\cdot -}$ in the composite coating group (53.6 μmol kg^−1^) was significantly lower than those in the other two groups. Meanwhile, the H_2_O_2_ contents in all groups showed single-peak curved changes during storage, and peaks were observed on day 6 (Figure 4D). On day 15, the H_2_O_2_ content in the control group (0.29 mmol kg^−1^) was significantly higher than those in the coated groups. Taken together, the composite coating effectively reduced the accumulation of ROS, and the reason for this appears interesting.

### 3.6. Antioxidant Enzyme Activities

**Figure 5:**
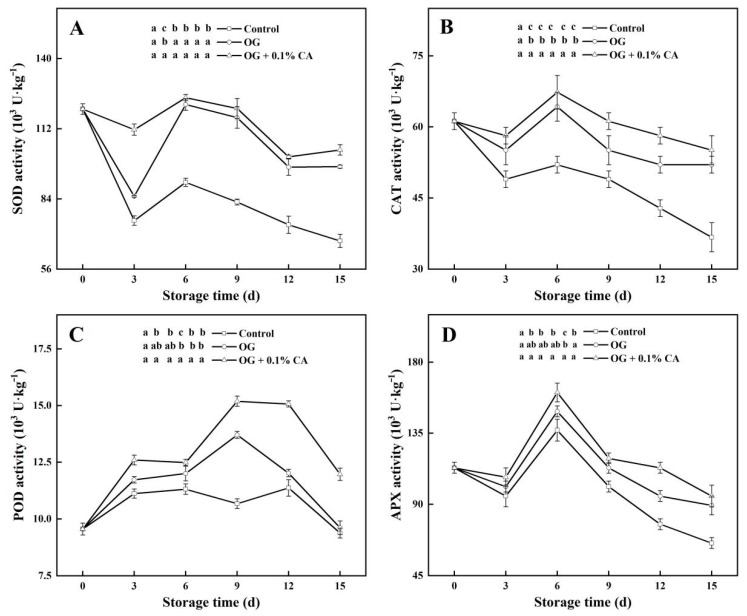
When stored at 25 °C and 85~95% RH, the cherry tomatoes with composite coating of OG and 0.1% CA kept higher activities of antioxidant enzyme SOD (**A**), CAT (**B**), POD (**C**), and APX (**D**) than the control fruits and the fruits with plain coating of OG. Bars represent standard errors (±SE). Different lowercase letters mean statistically significant differences (*p* < 0.05).

The activities of SOD, CAT, POD, and APX were elevated by the coatings; nonetheless, slight perturbation in the rhythms of the enzyme activity variations over time were observed. The fruits with the composite coating always showed the highest activities of these enzymes during storage, followed by the fruits with the plain coating and then by the fruits without a coating (Figure 5). Notably, the change rhythm of the POD activity differed from those of the other three enzymes. The POD activities in both coated groups first peaked at around day 9, but the fruits with the composite coating maintained their peak levels until day 12 and then slumped in the same way as the plain-coated fruits after the peak on day 9. In contrast, the POD activity in the control fruits experienced no sharp increase and leveled down during late observation. Nevertheless, the fruits with the composite coating showed the highest POD activities among all fruits at most time points, especially after day 9 (Figure 5C).

Although the investigated enzyme activities decreased in a synchronized manner from the appearance of peaks onward, the fruits with the composite coating always showed the highest activities for all four enzymes at the end of observation. The SOD activity in the fruits with the composite coating was 103.55 U kg^−1^, 1.07- and 1.54-times those in the fruits with the plain coating and without a coating, respectively. The CAT activities in both coated groups were significantly higher than in the control group. Remarkably, the POD activity in the fruits with the composite coating was the highest (11.97 U kg^−1^), followed by those with the plain coating (9.60 U kg^−1^) and without a coating (9.37 U kg^−1^). As was the situation for CAT, the highest APX activity was still recorded in the fruits with the composite coating (95.25 U kg^−1^), while the value in the control group was significantly lower (65.49 U kg^−1^). Collectively, the application of the composite coating potentiated the antioxidant enzyme activities in the cherry tomatoes during storage.

### 3.7. AsA and GSH Contents

**Figure 6:**
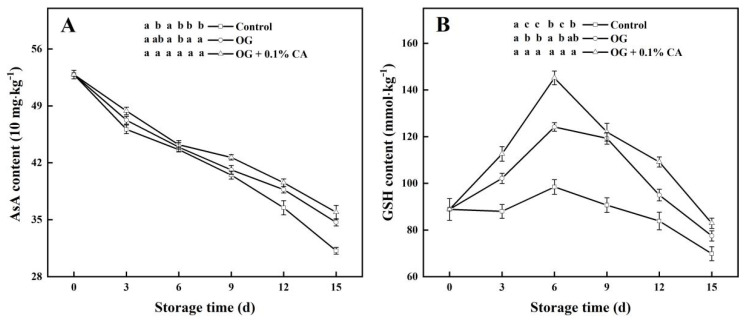
When stored at 25 °C and 85~95% RH, the cherry tomatoes with composite coating of OG and 0.1% CA showed higher levels of AsA (**A**) and GSH (**B**) than the control fruits and the fruits with plain coating of OG. Bars represent standard errors (±SE). Different lowercase letters mean statistically significant differences (*p* < 0.05).

Apart from enzymatic machinery, ROS can also be removed by multiple nonenzymatic components such as AsA and GSH, working by donating electrons to the ROS molecules [39]. Here, the AsA contents in all fruits steadily reduced during storage (Figure 6A), but the coated fruits always kept a relatively higher AsA content, especially in the late period. On day 15, the AsA content in the composite coating group was significantly higher than those in the other two groups. Additionally, the GSH contents in all fruit increased over the first 6 d of storage to peaks and then decreased (Figure 6B). The fruits with the composite coating possessed the highest GSH content (82.89 mmol kg^−1^) on day 15, followed by the plain coating group and then the control group. Altogether, both coatings effectively retarded the decreases in the AsA and GSH contents, more notably the composite one, thereby conserving the antioxidant capacity of the fruits during storage.

## 4. Discussion

The postharvest deterioration of cherry tomatoes is fast due to their perishable nature, resulting in susceptibility to pathogens and physiological decay [7]. Herein, both the plain and composite coating treatments effectively decreased the decay and preserved the marketability of the fruits during storage, wherein the composite coatings were more efficacious than the plain coating without CA. Predominantly, this difference was attributed to the involvement of CA, demonstrating antimicrobial and antioxidant activities [40], in the fruits’ protection. Composite coatings containing essential oil components curb the growth of surface microorganisms, thereby inhibiting the decay and maintaining the overall quality of the fruit [41]. A reported illustration of this point is the composite coating of tomato fruits with pectin incorporated with oregano essential oil [42]. A study published by our team demonstrated the effectiveness of CA in inhibiting microbial growth, reducing decay, and maintaining quality attributes of pears during storage [43]. Insights into the antimicrobial and antioxidant properties of CA reinforce our findings regarding its efficacy. Incorporating CA or similar compounds into coatings offers a sustainable solution for prolonging fruit shelf life and reducing postharvest losses [22].

Postharvest fruits continuously consume their nutrients to support cellular respiration for energy during storage. A higher rate of respiration consumes larger amounts of nutrients, thus begetting the fast maturing, aging, and softening and the easy decaying of the fruit [44]. Tomato fruits experience rapid weight reduction and softening owing to the water loss mainly caused by transpiration and high respiration rates, bringing on the loss of firmness of the fruit flesh [45]. Herein, the composite coating significantly inhibited respiration, slowing down the physiological metabolism and thus reducing the weight loss and retaining the firmness of tomato fruits. This effect is owing to the suppression of gas exchange through the fruit surface by the coating with specific polysaccharides and the increased fruit surface hydrophobicity as a result of the incorporation of lipophilic compounds into the coating. Coatings minimize fruits’ transpiration and respiration partly by covering their microporosities, cracks, or injuries and limiting the gas exchange of O_2_ and CO_2_, thereby decreasing the loss of fruit water and firmness [46]. Similar effects and their underpinning mechanisms on the coated fruits of strawberry [12], sweet cherry [47], guava [48], and mango [49] have previously been reported. While the respiration and transpiration rates and physiological ripening slow down, the fruit quality and sensorial property losses are mitigated [50].

The TA, TSS, and soluble protein levels reflect a fruit’s maturity and senescence state. TA, mainly on account of organic acids, accumulates during the fruit development stage and then is consumed as respiratory substrates in central carbon metabolism [51] during the ripening and senescence of the fruit [52]. Organic acids such as citric and malic acids are the principal substrates involved in the respiration of climacteric fruits [6]; thus, postharvest depletion of acidity is expected in fruits with high respiration rates. Variations in a fruit’s TSS content following harvest are closely related to both the hydrolysis of polysaccharides during ripening and the respiratory consumption of these hydrolysates [53]. During storage, the conversion of pectic substances, starch, hemicellulose, and other polysaccharides into soluble sugars contributes to the initial increase in the TSS content. This process leads to the accumulation of soluble sugars in the fruit, resulting in higher TSS levels [54]. However, despite the protective effects of a coating, metabolic processes within the fruit continue during storage. Over time, enzymatic activities and biochemical reactions may break down and convert sugars, potentially leading to a decrease in TSS levels [55]. The soluble proteins in fruit are mainly enzymes involved in various metabolisms and stress responses. A reduction in the soluble protein content is mainly attributed to the oxidation of certain proteins during storage, resulting in the inactivation of related enzymes and the accumulation of less soluble proteins in tissues [56]. Herein, the composite coating delayed the declines in the TSS, TA, and soluble protein levels, largely owing to the reduction in respiration. A coating suppresses respiration and thus slows the synthesis and degradation of metabolites, hence reducing nutrient consumption [27,57]. Accordingly, gum coatings of strawberry [12], sweet cherry [58], and fresh-cut apricot [59] maintain high levels of TSSs, TA, and soluble proteins in the fruit tissues.

The senescence of postharvest fruit is closely related to the abnormal accumulation of ROS. Under favorable circumstances, the generation and elimination of ROS achieves homeostasis, and the intracellular ROS are maintained at low levels, thus curbing the oxidative damage to cellular components and the formation of toxic products [33]. Postharvest fruit, however, experience multiple stresses from both environmental cues and internal physiological happenings, and these detrimental stresses vitiate the ROS homeostasis. The resultant overaccumulation of ROS accelerates the progress of fruit senescence [39]. Water loss, as a typical physiological stress, causes the excessive formation of ROS; the consequent oxidative damage, usually characterized by pronounced membrane lipid peroxidation and compromised membrane integrity, occasions metabolic disorders and further accelerates the quality deterioration and senescence of the postharvest fruit [28,57]. Herein, the composite coating effectively decreased the accumulation of $O_{2}^{\cdot -}$ and H_2_O_2_ and mitigated the elevation of cell membrane permeability. To improve the antioxidant efficacy of fruits, certain bioactive compounds such as plant polysaccharides and essential oils have been incorporated into coatings. Such incorporations enrich the protection of fruits against membrane lipid peroxidation, membrane integrity destruction, and fast senescence [60,61,62].

Endogenously, postharvest fruits deploy enzymatic and non-enzymatic arsenals to scavenge ROS and mitigate oxidative damage to cells [63]. The antioxidant enzyme machinery transforms ROS into less detrimental metabolites. Typically, SOD converts the more reactive $O_{2}^{\cdot -}$ through disproportionation reaction into H_2_O_2_, and this intermediate is then further converted into innocuous H_2_O and O_2_ by CAT, POD, and APX, collaboratively [64]. Herein, the composite coating potentiated the enzymatic antioxidant machinery of the fruits, as evidenced by the increased activities of SOD, CAT, POD, and APX. This potentiation was collaboratively underpinned by the performance of the bioactive components of the composite coating. The application of citral induces systemic acquired resistance in plants against pathogens [65], the cornerstone of which is the elevation in antioxidant gene expression to remove the extra ROS [66]. Furthermore, the antioxidant activities of CA and OG per se quench part of the fruits’ ROS, thereby reducing the oxidative stress on the fruits and retarding their senescence [67,68]. Accordingly, the composite coatings effectively preserved the overall quality of the cherry tomato fruits. Consistent with our findings, a Tragacanth gum coating regulated oxidative stress in postharvest apricots and therefore kept the quality of the fruits [13].

Non-enzymatically, endogenous antioxidant substances play crucial roles in scavenging fruit ROS. Among them, AsA and GSH in the versatile ascorbate–glutathione cycle are the dominant ones; the levels of these pivotal molecules are critical in maintaining the homeostasis of the intracellular antioxidant system and, thus, in alleviating the oxidative damage to fruit cells [69]. Herein, the fruits with the composite coatings kept higher levels of AsA and GSH than their counterparts in the control group; higher levels of AsA and GSH consolidate a more effective defense against various oxidative attacks. Taken together, the composite coatings reinforced both the enzymatic and non-enzymatic antioxidant machineries to alleviate stresses, retarded the fruits’ senescence, and thus preserved their quality.

## 5. Conclusions

The composite coatings of OG and CA effectively reduced the postharvest decay and maintained the marketability of cherry tomatoes stored at 25 °C and 85~95% RH. The coating with OG + 0.1% CA reduced the weight loss, respiration rate, and softening of the fruits and decelerated the decreases in their total soluble solid, titratable acidity, and soluble protein levels. Furthermore, the coating reduced the accumulation of $O_{2}^{\cdot -}$ and H_2_O_2_, and thus decreased MDA production and cell membrane permeabilization, mitigating the oxidative damage to the cells and, therefore, retarding the fruits’ senescence. Concomitantly, and as the underpinning mechanisms, the composite coatings elevated the activities of SOD, CAT, POD, and APX and the levels of AsA and GSH. This potentiation of the fruits’ antioxidant system was largely attributed to the integration of the coating components, which actuated the fruits’ stress responses and the antioxidant activities of the components per se.

## Data Availability

The data presented in this study are available upon request from the corresponding author. The data are not publicly available due to privacy.
